# Spatial-Temporal Forecasting of Air Pollution in Saudi Arabian Cities Based on a Deep Learning Framework Enabled by AI

**DOI:** 10.3390/toxics13080682

**Published:** 2025-08-16

**Authors:** Rafat Zrieq, Souad Kamel, Faris Al-Hamazani, Sahbi Boubaker, Rozan Attili, Marcos J. Araúzo-Bravo

**Affiliations:** 1 Department of Public Health, College of Public Health and Health Informatics, University of Ha’il, Ha’il 55471, Saudi Arabia; r.zrieq@uoh.edu.sa; 2 Applied Science Research Center, Applied Science Private University, Amman 11937, Jordan; 3 Department of Computer & Network Engineering, College of Computer Science and Engineering, University of Jeddah, Jeddah 21959, Saudi Arabia; skamel@uj.edu.sa (S.K.); sboubaker@uj.edu.sa (S.B.); 4 Department of Health Informatics, College of Public Health and Health Informatics, University of Ha’il, Ha’il 55471, Saudi Arabia; f.alhamzani@uoh.edu.sa; 5 Department of Medical Laboratory Science, Faculty of Pharmacy and Medical Science, Hebron University, Hebron P700, Palestine; rozana@hebron.edu; 6 Computational Biology and Systems Biomedicine, Biogipuzkoa Health Research Institute, 20014 San Sebastian, Spain; 7 Basque Foundation for Science, IKERBASQUE, 48009 Bilbao, Spain; 8 Department of Cell Biology and Histology, Faculty of Medicine and Nursing, University of Basque Country (UPV/EHU), 48940 Leioa, Spain

**Keywords:** air pollution, health, quality of life, modeling, prediction, deep learning (DL), long short-term memory (LSTM), random forest (RF), eXtreme Gradient Boosting (XGBoost), Saudi Arabia

## Abstract

Air pollution is steadily increasing due to industrialization, economic activities, and transportation. High levels pose a significant threat to human health and well-being worldwide. Saudi Arabia is a growing country with air quality indices ranging from moderate to unhealthy. Although there are many monitoring stations distributed throughout the country, mathematical modeling of air pollution is still crucial for health and environmental decision-making. From this perspective, in this study, a data-driven approach based on pollutant records and a Deep Learning (DL) Long Short-Term Memory (LSTM) algorithm is carried out to perform temporal modeling of selected pollutants (PM_10_, PM_2.5_, CO and O_3_) based on time series combined with a spatial modeling focused on selected cities (Riyadh, Jeddah, Mecca, Rabigh, Abha, Dammam and Taif), covering ~48% of the total population of the country. The best forecasts were provided by LSTM in cases where the datasets used were of relatively large size. Numerically, the obtained performance metrics such as the coefficient of determination (R^2^) ranged from 0.2425 to 0.8073. The best LSTM results were compared to those provided by two ensemble methods, Random Forest (RF) and eXtreme Gradient Boosting (XGBoost), where the merits of LSTM were confirmed mainly in terms of its ability to capture hidden relationships. We also found that overall, meteorological factors showed a weak association with pollutant concentrations, with ambient temperature exerting a moderate influence. However, incorporating ambient temperature into LSTM models did not lead to a significant improvement in predictive accuracy. The developed approach can be used to support decision-making in environmental and health domains, as well as to monitor pollutant concentrations based on historical time series records.

## 1. Introduction

Air pollution is characterized by the presence of chemical and biological substances harmful to human health [1] and constitutes a major global public-health challenge [2,3]. It harms social, economic, and health systems, as well as having a significant impact on health, causing up to 7 million premature deaths annually worldwide [4]. The rise in air pollution has increased the risk of cardiovascular disease, acute and chronic lung diseases and lung cancer [5]. In addition, traffic-related air pollution, comprising a complex variety of particles, is the most dangerous form of pollution, particularly for the respiratory system [6]. In particular, recent evidence shows that fine particulate air pollution (PM_2.5_) is strongly associated with genomic alterations in lung cancer tumors among individuals who have never smoked [7]. About 25% of global lung cancer cases occur in never-smokers, with PM_2.5_ exposure being a major environmental driver through its link to specific mutational signatures and telomere shortening. These findings underscore the critical role of ambient air pollution in lung carcinogenesis among never-smokers and the need for targeted environmental prevention strategies.

Inhalation is the major route for airborne pollutants to enter the human body. Air pollutants are typically classified by their physical phase into two primary categories: gaseous and particulate matter (PM). Common gaseous pollutants include nitrogen dioxide (NO_2_), sulfur dioxide (SO_2_), ozone (O_3_), carbon monoxide (CO) and carbon dioxide (CO_2_), which are prevalent in both urban and industrial atmospheres. PM encompasses a heterogeneous mixture of solid and liquid particles suspended in air, characterized by a range of aerodynamic diameters. Trace heavy metals such as lead (Pb) and chromium (Cr) are predominantly associated with PM, either absorbed onto particle surfaces or integrated within their matrix, rather than existing in the free gaseous phase. PM enriched in toxic heavy metals, particularly Pb and Cr, is among the most harmful to human health and is often used as a reference indicator in air quality assessments [8].

PM is categorized according to its aerodynamic diameter into PM_10_ (≤10 µm) and PM_2.5_ (≤2.5 µm). PM_10_ originates mainly from mechanical processes like road dust, construction, and soil resuspension, and typically contains mineral fragments, biological debris, and coarse crustal elements. In contrast, PM_2.5_ comes mainly from combustion sources (e.g., car emissions, industrial fuel burning, and wildfires) and is chemically richer in sulfates, nitrates, ammonium, organic carbon, and transition metals [9]. Upon inhalation, these particles differ in deposition sites in the respiratory tract. Multiple path particle dosimetry (MPPD) modeling shows that most PM_10_ particles deposit in the upper airways, whereas PM_2.5_ particles penetrate more deeply in the lungs and can reach the bloodstream, making it more toxic [10]. In this context, cardiovascular impacts are well-documented. PM_2.5_ enters the bloodstream, inducing vascular inflammation and increasing the risk of hypertension, heart attack, and stroke. Based on a Korean report, each increase of 2.9 µg/m^3^ in PM_2.5_ was associated with a 11.6% increased incidence of cardiovascular events [11], consistent with American Heart Association findings [12].

Saudi Arabia’s population, ~34.6 million, is projected to reach ~37.4 million by 2030 and ~40 million by 2035 under United Nations medium-variant forecasts. This rapid growth is fueling urbanization and a surge in energy demand, especially in major cities like Riyadh, expected to grow from 7 million in 2022 to ~9.6 million by 2030 (38% increase). As urban populations expand, so do the indirect consequences for air quality. Higher electricity consumption, still largely dependent on fossil fuels, increases emissions of CO_2_, NOₓ, and particulates. Additionally, rising numbers of private vehicles, buses, and freight traffic intensify pollution from nitrogen oxides and PM_2.5_, while growing industrial and construction activities further contribute to deteriorating air quality. Though population growth is not a direct pollutant source, it is a strong indirect source, accounting for ~38.7% of energy-related CO_2_, by amplifying energy use, transportation, and industrial development. This environmental impact challenges Saudi Arabia’s Vision 2030, which emphasizes environmental preservation and improved quality of life. However, as economic and industrial expansion and increasing vehicle use continue to elevate pollution levels, it becomes essential to prioritize the investigation and modeling of key pollution indicators. Spatial and temporal modeling can enhance understanding of the dynamic behavior of these pollutants, enabling the development of effective planning tools to mitigate the adverse effects of air pollution and support sustainable urban environments.

Given the global challenges of air pollution, researchers have conducted numerous studies modeling air pollution indicators across various locations and time periods, using diverse algorithms. Results vary with context—study location, datasets, methods, and computational resources. While classical prediction tools, such as deterministic and statistical methods, can provide reasonable forecasts, there is a growing trend toward the use of artificial intelligence (AI), owing to its superior ability to model complex, nonlinear relationships that are difficult to capture with traditional forecasting techniques. Machine learning (ML) and deep learning (DL) have received particular attention because of their potential to handle large amounts of data and to uncover hidden features. Moreover, the rapid growth of high-performance computers (HPCs), computer clusters, and specialized software packages dedicated to time-series forecasting and modeling has contributed to the development of the pollution control field.

To support health decision-making, modeling and monitoring pollutant concentrations such as PM_10_, the predictability of those concentrations using a deep convolutional neural network (CNN) has been explored [13]. The findings of the proposed framework were validated using a time series of hourly PM_10_ observations spanning the years 2000 to 2018 in Mexico City. An improvement of 20% compared to the classical Multi-Layer Perceptron (MLP) algorithm was reported. Through the same study, the concept of hybrid models involving more than one technique was also investigated. The results obtained with a combined model including a deep CNN and an ensemble algorithm were found to outperform individual methods considered separately from a spatial perspective. The Iranian city of Ahvaz in Khuzestan is ranked among the most polluted cities in the world due to severe dust storms generating high amounts of pollutants. To study the phenomenon of frequent pollution in this city, ref. [14] assessed the predictability of air pollutants using an artificial neural network (ANN) and highlighted the importance of developing additional frameworks to improve forecasting accuracy, support decision-making, and mitigate the negative impact of pollution. Other case studies for modeling PM_10_ and PM_2.5_ were considered in Poland over various cities. The results obtained in [15] were found to be of variable levels of accuracy mainly when considering climatic conditions and human activity indexes as explanatory variables. Based on observations and measured air quality indicators, the study in [16] examined the air quality in Saudi Arabia’s NEOM, a large-scale planned region in northwestern Saudi Arabia. However, a number of elements that could have an impact on the models’ quality were not considered, including the impact of vehicle traffic and industrial operations. According to a different study carried out in Malaysia [17], using ANNs in conjunction with principal component analysis (PCA) is an effective approach to model air quality, achieving an coefficient of determination (R^2^) of 0.68. Statistical and DL methods have been used to investigate long-term PM_10_ and PM_2.5_ prediction in Kolkata, India [18]. Since DL algorithms require extensive data, statistical approaches have produced superior results. The efficacy of multiple forecasting models based on various meteorological covariates (explanatory factors) was examined in a study carried out in the holy city of Mecca, Saudi Arabia [19]. The Multiple Linear Regression (MLR) model was found to outperform the other models investigated in predicting hourly PM_10_. The study carried out in [20] estimated the air quality index for the same place (Mecca City) using satellite remote sensing data. Mecca, Mina and Arafa were studied as the locations in which Muslim pilgrims perform Hajj. It was shown that PM_10_ increases during Hajj in these three locations. Other interesting works on modeling the pollution index have been conducted using fuzzy and statistical time series, respectively, in Kuala Lumpur, Malaysia [21], ML algorithms in Barcelona, Spain [22] and Ankara, Turkey [23], dispersion models in Mecca City, Saudi Arabia [24] and DL in Seoul, South Korea [25].

Due to the high sensitivity of the prediction task to the study location, the datasets used, and the techniques and methods employed, the results were sometimes found to differ even though the same conditions were used to run the prediction algorithms. Table 1 below summarizes selected studies while focusing on the pollution indicators, location, results, and limitations. This in-depth analysis may help situate the contributions of our study.

The reviewed literature (particularly the references included in Table 1) highlights the global severity of air pollution and its increasing threat to human health and environmental sustainability across continents. Among the wide range of pollutants studied, PM_10_, PM_2.5_, and NO remain the most frequently analyzed due to their direct health implications, while others, such as CO and NO_2_, have also gained research attention. Across the studies, model performance and predictive accuracy were shown to be highly case-sensitive, varying not only with the chosen ML or DL technique but also with pollutant type, dataset size, time resolution, and data quality. A consistent observation is that DL models, particularly the Long Short-Term Memory (LSTM) architecture, tend to outperform traditional ML models in capturing temporal patterns when sufficient historical data are available. However, challenges such as hyperparameter tuning, missing data handling, and model generalization remain common across AI-based approaches. LSTM [34] was chosen over ANN approaches such as Gated Recurrent Unit (GRU) and Recurrent Neural Networks (RNNs) for modeling/predicting time series data of pollution variables is because it explicitly captures long-term dependencies in time series data and quantifies the effect of past values on current and future ones. In our pollution prediction, external factors such as temperature, relative humidity, atmospheric pressure, wind speed, and wind direction are considered to implicitly affect the pollution indicators through their past values since their effects are inherently propagated through the LSTM units. LSTM is therefore considered to be more effective than RNNs and GRUs in solving problems related to the vanishing gradient problem, which is usually encountered when training these DL algorithms. Moreover, RNNs often lose the ability to retain information from earlier time steps in the sequences. This problem is overcome using the LSTM architecture, which introduces a cell state and specialized gates (forget, input, and output) that enable the model to selectively retain or discard information over long periods. This capability is crucial for accurate prediction, especially with datasets exhibiting complex seasonality or trends.

In response, our study adopts LSTM as a core forecasting framework, while explicitly addressing known limitations through careful data preprocessing and model design. Moreover, to ensure the robustness of our approach, we conduct a comparative evaluation against ensemble-based methods (RF and XGBoost) and apply the models across a case-sensitive spatio-temporal setting focused on key cities in Saudi Arabia. This allows for a more context-aware and performance-driven assessment of air pollution forecasting models. A more explicit justification of what distinguishes this work from the existing literature can be summarized as follows:

- Unique Spatial Coverage: Our study involves seven major cities across Saudi Arabia (Riyadh, Jeddah, Mecca, Rabigh, Abha, Dammam, and Taif), covering ~48% of the total population of the country. To the best of our knowledge, this is one of the most comprehensive spatial analyses of air pollution using deep learning in the Gulf region, and particularly within Saudi Arabia.
- Spatial-Temporal Modeling Integration: While many studies focus solely on temporal forecasting or single-city analysis, our work integrates both spatial and temporal modeling across a wide geographical region, allowing for regional comparisons and more generalizable insights.
- Model Benchmarking with Ensemble Learning: We perform a comparative evaluation between LSTM, RF, and XGBoost to demonstrate the relative strengths and weaknesses across varying data scenarios. This comprehensive benchmarking across DL and ML models adds robustness to our conclusions.
- Investigation of Meteorological Influence: The study includes a detailed analysis of meteorological variables (temperature, humidity, pressure, wind speed, and wind direction), revealing that most of them show weak correlation with pollutant levels, except temperature. This adds a context-specific insight for forecasting in arid climates, which is underrepresented in current literature.
- Findings on LSTM Data Sensitivity and Feature Importance: We observed and reported that LSTM performance improves significantly with larger datasets, reaffirming its suitability for urban settings with dense monitoring. Additionally, our results showed that adding temperature data did not significantly improve LSTM accuracy, which supports the efficiency of historical data-only models in similar contexts.
- Practical Implications for Data-Sparse Environments: Based on our results, we propose practical forecasting strategies for cities with limited meteorological data or sparse monitoring networks, making this work especially relevant for developing smart monitoring strategies in the Gulf and similar arid regions.

The remainder of this article is structured as follows: The Methods section details data collection, AI algorithms, and performance metrics. The Results section highlights key findings: variation in LSTM performance across pollutants and cities; station data volume as a key accuracy driver; correlations between LSTM performance, data volume, and urban density; weak meteorological–pollutant links with moderate temperature influence; limited impact of adding temperature to LSTM models; and comparison of LSTM with RF and XGBoost. The Discussion and Conclusions sections interpret the results, discuss implications, and suggest future research directions.

## 2. Methods

### 2.1. Study Area

**Figure 1:**
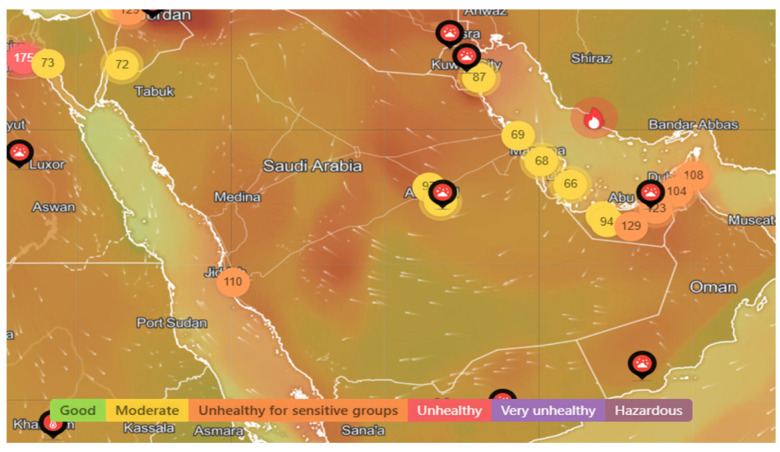
Example of a pollution distribution heat map of Saudi Arabia: the white arrows indicate the wind intensity and direction; the circles indicate the PM_2.5_ concentration (μg/m^3^). Good in green 5.1 ≤ PM_2.5_ ≤ 10, moderate in yellow 10.1 ≤ PM_2.5_ ≤ 15, unhealthy for sensitive groups in orange 15.1 ≤ PM_2.5_ ≤ 25, unhealthy in red 25.1 ≤ PM_2.5_ ≤ 35, very unhealthy in magenta 35.1 ≤ PM_2.5_ ≤ 50, hazardous in purple 50 < PM_2.5_.

Saudi Arabia is a West Asian country that covers most of the Arabian Peninsula and has a land area of ~2,150,000 km^2^, making it the fifth largest country in Asia. Saudi Arabia has a total population of ~32,175,224 inhabitants and a real Gross Domestic Product (GDP) growth rate of 2.8% as of the fourth quarter of 2024. The country has an Industrial Production Index (IPI) of 1% as of 1 August 2024 (General Authority for Statistics: https://www.stats.gov.sa/en#). Saudi Arabia is characterized by a desert climate, except for the southwestern part of the country, which has a semi-arid climate. Summers in the central region are extremely hot and dry, ranging from 27 °C to 43 °C in the inland areas and 27 °C to 38 °C in coastal areas (World Bank Climate Change Knowledge Portal: https://climateknowledgeportal.worldbank.org). There are more than 6,000,000 cars in Saudi Arabia, as personal cars are the preferred means of transportation for the vast majority of people living in the country. As per Figure 1 and according to the Air Quality Index website (https://www.iqair.com/sa/saudi-arabia, accessed 12 December 2024), the pollution index ranges between moderate and unhealthy, despite the discrepancy between Saudi cities.

### 2.2. Data Collection

To carry out this study, the datasets used to model air pollution were collected from two official sources as follows:

(i) National Center for Environmental Compliance Control (NCECC) (https://ncec.gov.sa/, accessed 17 April 2025), which was previously part of the activities of the Meteorology and Environmental Protection Authority (MEPA) (currently, National Center for Meteorology (NCM) (https://ncm.gov.sa/, accessed 17 April 2025).
(ii) KAPSARC School of public policy (https://www.kspp.edu.sa/, accessed 17 April 2024): Via its portal, this school published a dataset that contains Riyadh air quality for 2019–2020. The data are reported to be collected by KAPSARC from The General Authority of Meteorology & Environmental Protection. The dataset is available at: https://datasource.kapsarc.org/explore/dataset/air-quality/ (accessed on 17 April 2025).

**Figure 2:**
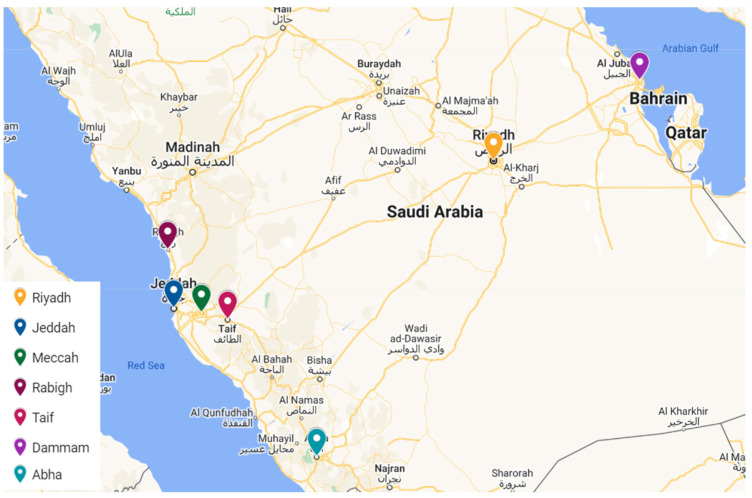
Locations of selected Saudi cities for pollution data collection, shown with decimal degree coordinates in the format (latitude, longitude): Abha (18.22° N, 42.51° E), Dammam (26.33° N, 43.97° E), Jeddah (21.54° N, 39.20° E), Mecca (21.43° N, 39.83° E), Rabigh (22.80° N, 39.03° E), Riyadh (24.69° N, 46.72° E), and Taif (21.27° N, 40.42° E). Source: SDAIA, Saudi Open Data Portal.

It should be noted here that all the datasets were collected from NCECC except for the Riyadh datasets, which were collected from KAPSARC. The collected data cover different time periods and selected cities with different characteristics. The selected cities are the following: Riyadh, Jeddah, Mecca, Rabigh, Taif, Abha, and Dammam (Figure 2). These cities represent the main characteristics related to the pollution distribution such as location, local climate, population, number of cars and economic/industrial activities as well as meteorological parameters. Regarding the population of these cities, Riyadh with 7,009,100, ranks 1st (2022), Jeddah with 3,751,722 ranks 2nd (2022), Mecca with 2,385,509 ranks 3rd (2022), Rabigh with 180,352 (2014), Abha with 334,290 (2022), and Dammam with 1,386,671 ranks 5th, based on data from the portal.saudicensus.sa (in Arabic), together covering ~48% of the total population of Saudi Arabia and therefore counting as a highly valuable representation of the impact of pollution across the whole country. These figures are approximate and may vary slightly based on the most recent data available. Saudi Arabia’s population is growing rapidly, so these rankings may change with new census data.

Regarding the dataset size and temporal coverage described in Table 2, the following remarks are noteworthy:

- Data availability constraints: The raw pollution datasets were obtained from various official monitoring stations across Saudi Arabia, and not all stations provided continuous or complete data for the same years. Some datasets were limited by missing values, instrument outages, or gaps in reporting.
- Case-sensitive analysis: We aimed to conduct a spatially distributed analysis covering multiple urban contexts rather than focusing solely on a single city. While this resulted in different time windows, it offered insight into the performance of forecasting models under varied local conditions (e.g., coastal vs. inland cities, industrial vs. residential zones).
- Data quality filtering: To ensure meaningful training, we filtered out sequences with excessive missing values or poor data quality, which reduced the usable time span in some cases.

The collected datasets (Table 2) were primarily visualized to detect any irregularities, such as missing values or anomalously high or low outliers, given that the pollution monitoring stations are installed in remote locations with harsh climatic conditions, which may result in erroneous readings. Missing values were imputed based on the assumption that the subsequent value is equal to the previous one, considering that changes in pollution indicators typically occur gradually. However, in cases where the number of missing values was high, this imputation method was not applicable. Instead, the available datasets were screened to identify relatively complete subsets, allowing for at most two consecutive missing values. Furthermore, since this study aims to evaluate the performance of the LSTM deep learning algorithm in modeling air pollution in Saudi Arabia, even limited-size datasets were utilized, although LSTM models generally require large amounts of data to perform optimally.

### 2.3. General Flowchart of Study

We used the LSTM architecture, along with the RF and XGBoost ensemble methods (for comparison), to model and forecast air pollution indicators in selected cities across Saudi Arabia. The core concept of time series modeling lies in using sequential historical records of the pollutant variable (e.g., PM_10_, PM_2.5_, CO, or O_3_)—denoted as *y*—spanning a fixed number of time steps (referred to as *n* steps, set to five based on established best practices). In our implementation, this corresponds to an input vector comprising the previous five observations (hours or days depending on the available data source). The output is the predicted value of the same pollutant at the next time step (i.e., a one-step-ahead forecast). Therefore, the inputs are defined as [*y*(*t* − 1), *y*(*t* − 2), …, *y*(*t* − *n*)], and the output is *y*(*t*). This sliding window is also used for the RF and XGBoost. When studying the effect of the ambient temperature AT as a climatic factor, the historical lags of the ambient temperature [AT(*t* − 1), AT(*t* − 2), …, T(*t* − *n*)] are used as inputs in addition to the historical lags of the concerned variable *y*. In our current implementation, we limited our analysis to short-term, one-step-ahead (1 h/1 day) forecasting, which is standard in air quality prediction and relevant for near-real-time applications. This decision was made to maintain consistency across models and pollutants and to ensure that error metrics reflect direct and interpretable performance.

It is important to note that in our LSTM-based framework, historical PM values served as the primary input features. These historical values implicitly capture the combined effects of various factors contributing to PM concentrations—such as emissions from transportation, industry, and domestic sources, as well as regulatory or behavioral changes over time. Due to the recurrent memory structure of LSTM networks, these influences are retained and propagated through time steps, allowing the model to learn complex temporal patterns without requiring all causal variables explicitly provided as inputs.

Although meteorological variables individually contributed modestly to model performance in certain scenarios, they were retained given their established role in short-term PM fluctuations, affecting dispersion, chemical transformation, and deposition processes.

Additionally, socioeconomic indicators (e.g., income levels, industrial development) and population density tend to evolve slowly over time and primarily influence long-term trends in air pollution. Since the current study focused on short-term forecasting, such variables may show minimal variability within the forecasting window and are therefore less impactful in enhancing immediate predictive performance. Nevertheless, for future studies focused on long-term forecasting or policy analysis, the integration of explicit emission inventories and urban development metrics could provide additional insights and improve model interpretability.

**Figure 3:**
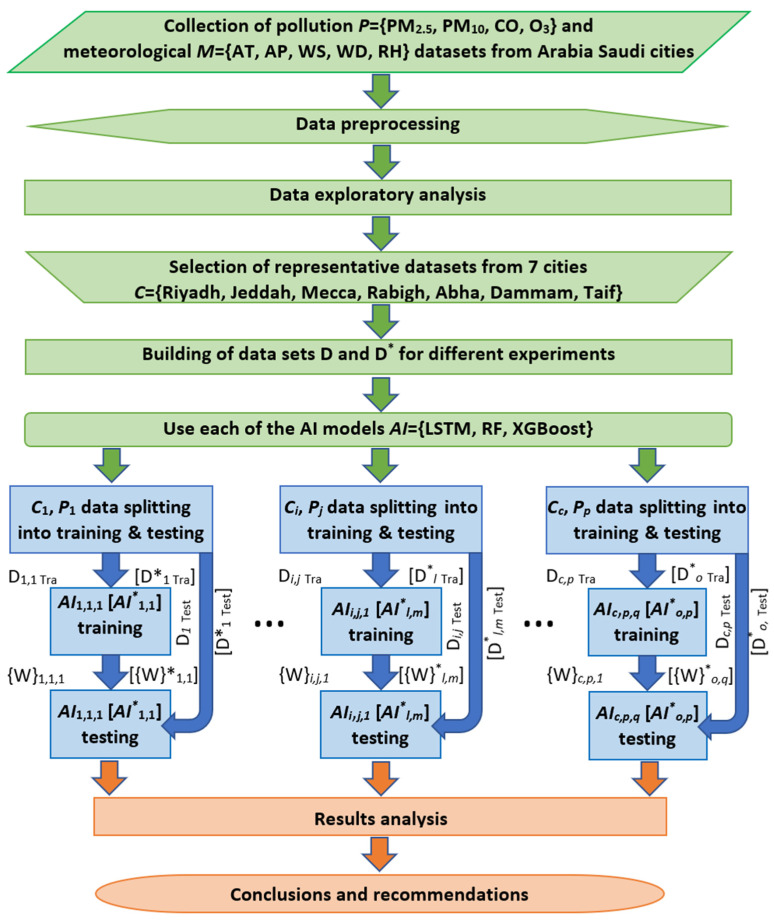
Study flowchart. The green, blue and orange blocks mark preprocessing, processing and postprocessing stages. *C*_1_ … *C_c_* represents the *c* = 7 selected cities *C* = {Riyadh, Jeddah, Mecca, Rabigh, Abha, Dammam, Taif} and *P*_1_ … *P_p_* represents the *p* = 4 available pollutants *P* = {PM_2.5_, PM_10_, CO, O_3_}. For additional experiments, we use the data from the four best behaving datasets D^*^ = {PM_10_ Jeddah (2019), PM_2.5_ Jeddah (Dhahban station) (2022), CO Dammam (2019), O_3_ Jeddah (2018)}. To investigate whether the inclusion of the meteorological parameters is able to improve the forecasters’ accuracies, we also include the ambient temperature AT from the meteorological dataset *M* as an input in the datasets D*. To compare the results of the LSTM predictor with other AI predictors, we create a set of predictors AI = {LSTM, RF, XGBoost}, and we use them to evaluate the datasets D*. D*_i,j,_*_1 Tra_ and D*_i,j,_*_1 Test_ are the datasets for training and testing of the city *i* and pollutant *j* with the model 1 (LSTM). {W}*_i,j,_*_1_ is the set of all trained parameters of the LSTM of the city *i* and pollutant *j*. The datasets D*_l_ _Tra_ for training and testing D^*^_l_ _Test_, models AI*_l_,*_m_* and weights W*_l_,*_m_* for the additional experiments (AT study and ensemble methods comparison) are marked with a superscript asterisk inside the brackets.

The procedure followed includes several steps, namely, data collection and exploratory analysis, rationale for selecting the LSTM technique, model training, model validation, and performance analysis (Figure 3). For this aim, 15 datasets covering the four pollutants (PM_2.5_, PM_10_, CO and O_3_) were selected from the covered cities so that they overall represented the whole territory of Saudi Arabia. As the datasets were found to have several missing records, an imputation procedure was used to replace those missing data under the assumption of slow dynamics of the pollutants’ concentration variation. Note here that the LSTM algorithm was run separately for the 15 datasets in addition to four experiments considering the ambient temperature as the main meteorological factor impacting the air pollutants’ concentrations as well as for two ensemble methods, namely, the RF and the XGBoost for comparison purposes.

Although LSTM is an established DL technique, it remains one of the most effective tools for capturing hidden temporal dependencies in time-series data. In our study, we conducted a trial-and-error comparative analysis with other ML algorithms, including RF and XGBoost. Our methodology follows a structured and replicable process commonly adopted in forecasting studies, including data collection, preprocessing, model development, and validation. Furthermore, this study contributes a comprehensive spatial-temporal analysis across seven major Arabia Saudi cities, making it one of the few regionally focused DL studies of its kind. As such, it provides both practical insights and scientific contributions to the understanding and forecasting of air quality in arid urban environments.

### 2.4. LSTM Implementation

Before applying the LSTM, Gated Recurrent Unit (GRU) and Recurrent Neural Networks (RNNs), as well as their bidirectional versions (Bi-GRU and Bi-RNN), they were investigated based on the trial-and-error principle. To evaluate the quality of the models, several performance metrics such as mean absolute percentage error (MAPE) and coefficient of determination (R^2^) were calculated. In addition, a careful study was made of the reasons why authors of previous studies chose LSTM over GRU and RNN. LSTM was found to be a reasonable choice, ensuring a high level of compromise between accuracy and complexity. In the following, the LSTM technique is presented in detail.

In many real-world time-series forecasting problems, such as air pollution, climate data, or energy demand, the information can be highly noisy (due to measurement device biases) or volatile. LSTM’s more complex gating mechanisms allow it to better filter out irrelevant information and focus on important patterns unlike GRU and RNN. Because LSTM networks are a type of RNN designed to remember long-term dependencies and avoid problems such as vanishing gradients, they are widely used for sequential data such as time series of pollution indicators, as in our case. The architecture of an LSTM cell consists of several gates that control the flow of information: (i) Forget Gate that decides which information to discard; (ii) Input Gate that decides which information to store; (iii) Candidate Cell State that adds new information to the cell; (iv) Cell State Update that combines old cell state with new information; (v) Output Gate that decides what information to output.

**Figure 4:**
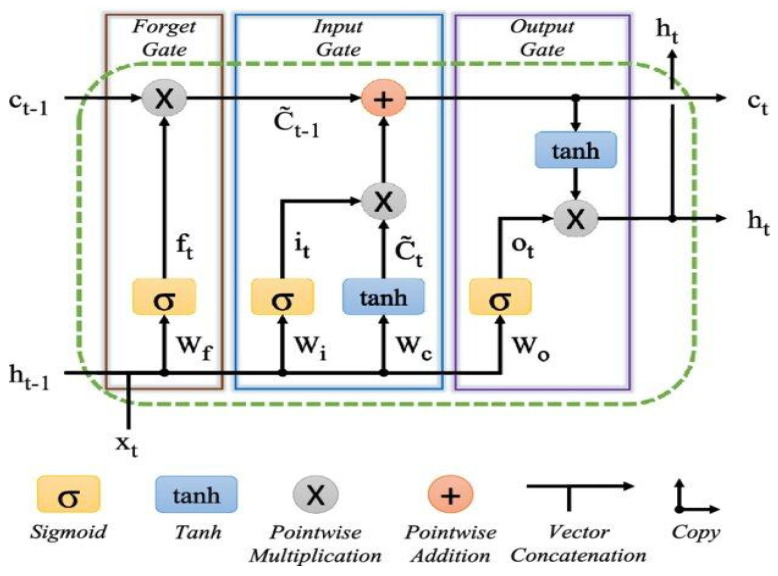
The basic structure of an LSTM architecture. Lower-case variables represent vectors. Matrices *W_q_* are the weights of the inputs, where the subscripts *q* are either the input *i*, output *o*, forget gates, or the memory cell *c*.

As shown in the basic structure of the LSTM (Figure 4) [35], the equations governing the operation of the LSTM are provided as follows:

(1) $$f_{t}=\sigma_{g}\left(W_{f}\cdot \left[h_{t-1},x_{t}\right]+b_{f}\right)$$

(2) $$i_{t}=\sigma_{g}\left(W_{i}\cdot \left[h_{t-1},x_{t}\right]+b_{i}\right)$$

(3) $$o_{t}=\sigma_{c}\left(W_{o}\cdot \left[h_{t-1},x_{t}\right]+b_{o}\right)$$

(4) $$c_{t}=f_{t}c_{t-1}+i_{t}\cdot tanh\left(W_{c}\cdot \left[h_{t-1},x_{t}\right]+b_{c}\right)$$

(5) $$h_{t}=O_{t}\cdot \sigma_{h}\left(c_{t}\right)$$

where $x_{t}\in R^{d}$ input to the LSTM unit, $f_{t}\in {\left(0,1\right)}^{h}$ forget gate’s activation, $i_{t}\in {\left(0,1\right)}^{h}$ input/update gate’s activation, $o_{t}\in {\left(0,1\right)}^{h}$ output gate’s activation, $h_{t}\in {\left(-1,1\right)}^{h}$ hidden state and $c_{t}\in R^{h}$ cell input activation are vector variables$;\;W_{f},\;W_{i},\;W_{o},\;W_{c}\in R^{h\times d}$ and $b_{f},b_{i},b_{o},\;b_{c}\in R^{h}$ are trainable weight matrices and bias vector parameters to be learned during the training phase; $\sigma_{g}$ sigmoid $\sigma_{c}$ hyperbolic tangent, $\sigma_{h}$ hyperbolic tangent, are activation functions. The superscripts *d* and *h* are the number of input features and hidden units, respectively, while the subscript *t* indicates the time step, and the initial values are *c*_0_ = *h*_0_ = 0.

Training an LSTM model involves using a loss function, typically cross-entropy or mean squared error for sequence prediction tasks. Backpropagation Through Time (BPTT) is used to calculate gradients. An optimizer, such as Adaptive Moment Estimation (Adam), is used to adjust the weights of deep neural networks. Adam computes adaptive learning rates for each parameter by maintaining running averages of both the gradients (first moment) and the squared gradients (second moment). These estimates help Adam converge faster and more reliably, especially in complex, high-dimensional spaces. Its ability to handle sparse gradients, noisy data, and non-stationary objective functions makes it a popular choice for training deep neural networks.

When using an LSTM deep neural network, hyperparameter tuning is essential for optimal architecture performance. Key hyperparameters for an LSTM include (i) Number of Layers: Determines the depth of the network; (ii) Hidden Units: The number of neurons in each layer; (iii) Learning Rate: Controls how fast the model adapts; (iv) Batch Size: Number of samples processed before model weights are updated; (v) Sequence Length: The number of time steps in each input sequence. Common hyperparameter tuning techniques include grid search, random search, trial-and-error, and Genetic Algorithm (GA).

### 2.5. Random Forest (RF) and Extreme Gradient Boosting (XGBoost) Implementation

RF [36] and XGBoost [37] are ensemble learning methods based on decision trees. However, they are known to differ fundamentally in how they build and combine trees. RF operates by constructing a large number of decision trees in parallel, each trained on a bootstrapped subset of the data with random feature selection at each split, and outputs the final prediction via majority vote (classification) or averaging (regression). Based on trial-and-error, its key hyperparameters are tuned in this study as follows: the number of trees (*n* estimators = 100), maximum tree depth (*max depth* = ‘default’), number of features considered at each split (*max features* = ‘auto’/‘sqrt’ (default)), and minimum samples required to split a node (*min samples split* = 2 (default)). In contrast, XGBoost builds trees sequentially using a boosting strategy that minimizes a regularized objective function through gradient descent. Each new tree corrects the residual errors of the previous ones, and the model incorporates both L1 and L2 regularization to reduce overfitting.

We also tuned by trial-and-error the XGBoost’s main hyperparameters: learning rate (0.1), number of trees (*n estimators* = 100), maximum tree depth (max depth = 4), subsampling ratios (*subsample* = 1), and regularization terms (α = 0, λ = 1). While RF is robust and easy to tune, XGBoost is often more accurate and computationally efficient for structured data tasks due to its boosting mechanism and optimization techniques.

### 2.6. Performance Metrics

The quality of the developed prediction models was measured by comparing the predicted values with the actual (reported) values. In this study, three performance metrics are calculated to evaluate the efficiency of the models. The performance metrics are expressed as follows [18,38]:

Mean Absolute Percentage Error (MAPE). Indicates the accuracy of the model’s predictions by measuring the average absolute percentage error between the predicted and actual values.

(6) $$MAPE=\frac{100}{N}\sum_{t=1}^{N}\frac{\left|y\left(t\right)-\hat{y}\left(t\right)\right|}{\underline{y}}$$

Coefficient of determination (R^2^). Represents the proportion of the variance in the dependent variable (pollutant concentration) that can be predicted by independent variables. It indicates how well the model fits the data, with values closer to 1 indicating a better fit.

(7) $$R^{2}=1-\frac{\frac{1}{N}\sum_{t=1}^{N}{\left(y\left(t\right)-\hat{y}\left(t\right)\right)}^{2}}{\frac{1}{N}\sum_{t=1}^{N}{\left(y\left(t\right)-\underline{y}\right)}^{2}}$$

Root Mean Square Error (RMSE). Measures the average magnitude of the error between predicted and actual values, with a lower RMSE indicating better model performance.

(8) $$RMSE=\sqrt{\frac{1}{N}\;\sum_{t=1}^{N}{\left(y\left(t\right)-\hat{y}\left(t\right)\right)}^{2}}$$

where $\hat{y}\left(t\right)$ and $y\left(t\right)$ represent the predicted and the actual (reported) values of the relevant pollution indicators (PM_10_, PM_2.5_, CO and O_3_), respectively, at time t. Note that time may refer to the hour or the day, depending on the monitoring station. $\underline{y}$ is the mean value of the dataset under evaluation. Lower MAPE and RMSE and higher R^2^ (value located between 0 and 1) are indicators of good data fitting [39]. *N* is the size of the dataset used. The performance metrics were calculated only for the testing/validation out-of-sample dataset (20%). It should be noticed here that out-of-sample dataset was not used during the training phase, and therefore, the LSTM, RF and XGBoost algorithms have no knowledge about it.

### 2.7. Hardware and Software Configuration

The resources used in terms of hardware were an Intel(R) Core (TM) i7-9750H CPU @ 2.60GHz microprocessor with 32.0 GB of installed RAM running on a 64-bit Windows 10 Operating System. In terms of software, TensorFlow/Keras (running through the Spyder (Python 3.8)/Anaconda environment) and Matlab®, version 10.0, R2020b (MathWorks, Natick, MA, USA) (for metrics calculation and figure plots generation) were used.

## 3. Results

### 3.1. LSTM Hyperparameter Tuning

As previously explained, LSTM [40] is a kind of deep RNN, whose main feature—compared to other basic DL architectures such as RNN and GRU—is its ability to include a long-term memory to keep information from historical values using the principle of gates (input, forget, output). Among the challenges of using LSTM is the selection of its hyperparameters, which must be carefully tuned to achieve the best accuracy of the derived predictions. Although there are several ways to tune LSTM hyperparameters, such as grid search, trial-and-error remains among the most commonly used techniques [41]. Therefore, in this study, trial-and-error was used after tens of runs with different hyperparameter settings. Table 3 summarizes the main hyperparameters of the LSTM. Note that the selected hyperparameters were kept unchanged for all experiments to allow for meaningful comparisons later on.

### 3.2. LSTM Performance Varies Significantly by Pollutant and City

Despite GRU and RNN, LSTM is known to be an excellent structure in terms of gradient explosion and vanishing problems. Based on the available datasets, 15 experiments were conducted to predict PM_10_, PM_2.5_, CO and O_3_ at seven locations over Saudi Arabia (Table 3). The efficiency of the conducted experiments was evaluated based on the MAPE, R^2^ and RMSE performance metrics. The performance metrics of the experiments performed are summarized in Table 4. We observe that LSTM performance metrics vary significantly depending on both the specific pollutant and the city under study. Since all LSTM hyperparameters were kept unchanged for all experiments, only the dataset size is considered as a factor that may affect the accuracy of the models.

### 3.3. Station Data Volume Drives Predictive Capability

**Figure 5:**
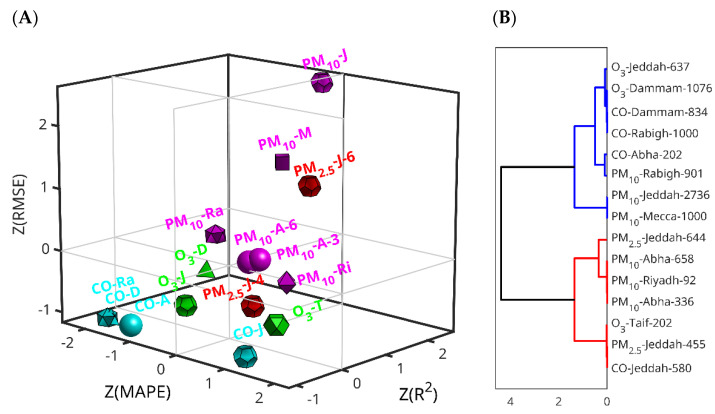
Analysis of the Z-scores of the three combined LSTM prediction metrics. (**A**) Three-dimensional scatter-plots of the three performance metrics. The simplified dataset coding is pollutant, first (and, in the case of Rabigh and Riyad, second letter of the city), plus the most significant digit of the dataset size when it is necessary to distinguish between cases of the same pollutant and city. The spheres, dodecahedra, cubes, octahedra, icosahedra, tetrahedra, and cube octahedra correspond to Abba, Jeddah, Mecca, Riyadh, Rabigh, Dammam and Taif, respectively; magenta, red, cyan and green correspond with PM_10_, PM_2.5_, CO and O_3_, respectively. (**B**) Hierarchical clustering of the three performances using the Euclidean distance and the Ward linkage method. The dataset coding is the pollutant-city-dataset size.

To investigate a possible relationship between the features of the sensor stations and the prediction performance of the LSTMs, we studied the three-dimensional position of the three metrics. A Z-score transformation was used to standardize the difference range of the metrics. The missing R^2^ value of PM_10_ of Riyadh (Al-Jazeera station) (2019–2020) was imputed using the mean of all R^2^ values of the remaining stations. The three-dimensional scatter plot shows that the CO predictions generally have negative Z-scores, meaning lower error (except for the case of Jeddah), the PM_10_ predictions are around Z-score zero (again except for the case of Jeddah), and the rest of the cases do not show such a clear pattern (Figure 5A). To have another view on this relationship, we performed hierarchical clustering using Euclidean distance and the Ward linkage method (Figure 5B). The hierarchical clustering reveals two main branches, one (in red) consisting of stations with higher numbers of data points (all above 600), and the other (in blue) comprising stations with fewer data points (all below 600), except for PM_10_ in Abha (with 658) and PM_2.5_ in Jeddah (with 644). This highlights that the amount of data generated by each station—and the corresponding 80% used for training—is a key factor influencing the predictive performance of the LSTM model.

### 3.4. LSTM Performance Strongly Correlates with Data Quantity and Urban City Density

**Figure 6:**
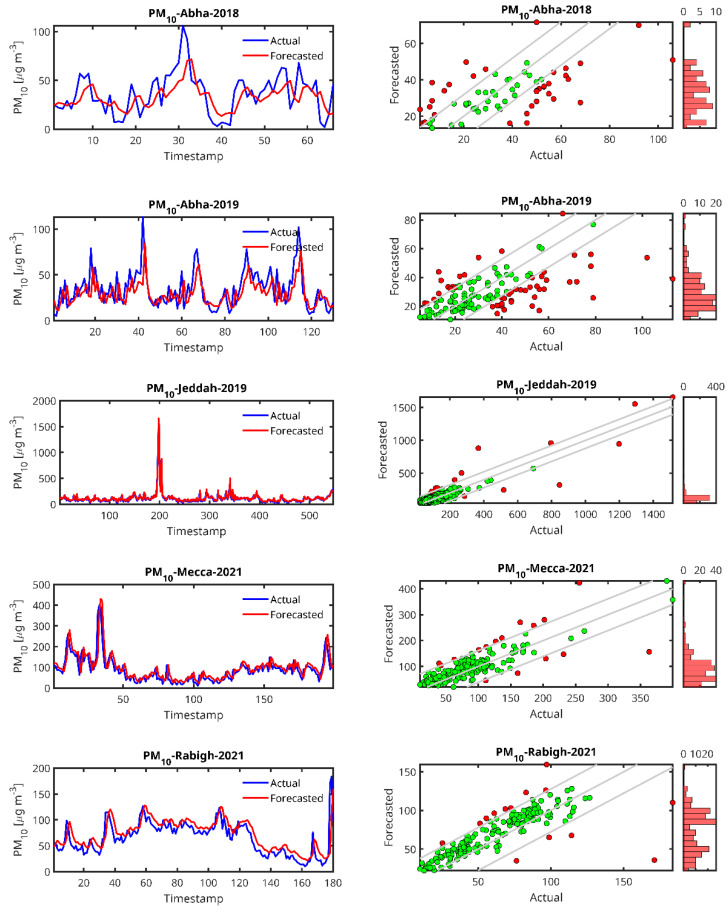

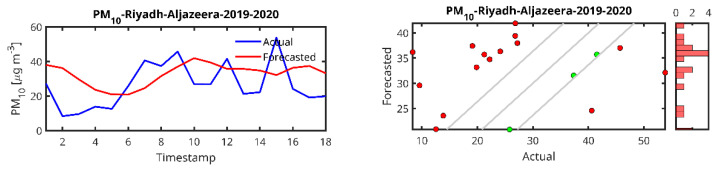
Actual and forecasted values for PM_10_. (**Left** panels) Time series profiles comparing the actual PM_10_ measured concentrations (blue) and the corresponding Forecasted values (red) over the evaluation period. (**Right** panels) Scatter plots of the forecasted PM_10_ values vs. the actual measurements. The central diagonal line represents the ideal 1:1 correspondence between forecasted and actual values. The upper and lower diagonals indicate the boundaries of ±σ_Forecasted_ around the actual values, where σ_Forecasted_ denotes the standard deviation of the forecasted values. Points marked in red indicate forecasted values deviating from the actual measurements by more than σ_Forecasted_, while green points represent values falling below the actual measurements by more than σ_Forecasted_. Adjacent to the scatter plots, histograms show the distribution of the forecasted PM_10_ values, offering insight into the model’s output spread and potential bias.

**Figure 7:**
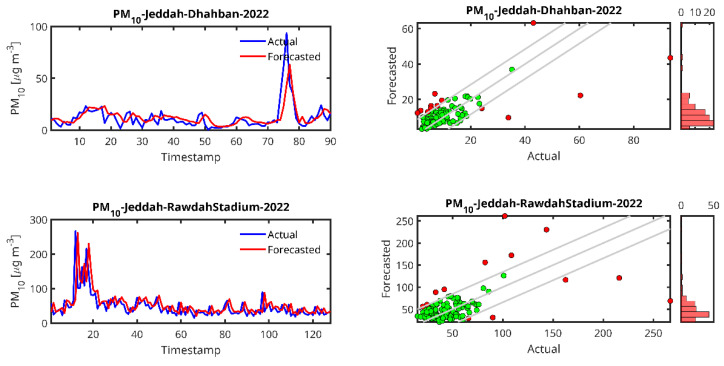
Actual and forecasted values for PM_2.5_ concentrations. Panels are structured identically to those in Figure 6, showing (**Left**) time series plots of actual (blue) and forecasted (red) PM_2.5_ values, and (**Right**) scatter plots comparing forecasted versus actual PM_2.5_ measurements. The diagonal lines, color coding, and accompanying histograms follow the same conventions as described in Figure 6, with σ_Forecasted_ representing the standard deviation of the forecasted PM_2.5_ values.

**Figure 8:**
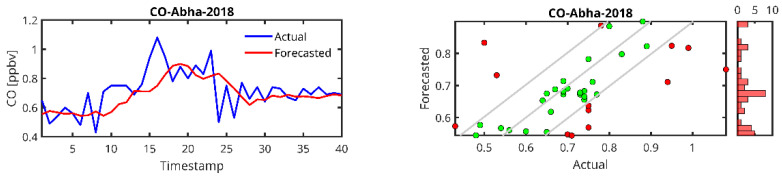

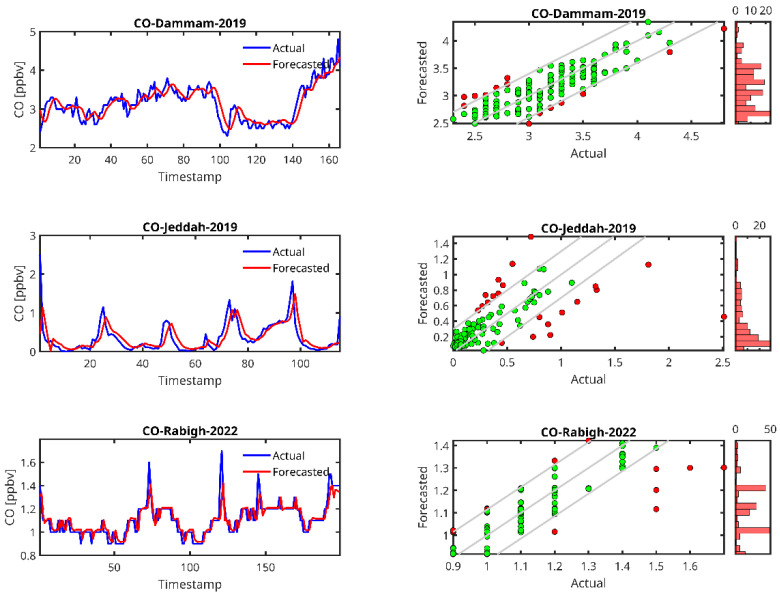
Actual and forecasted values for CO concentrations. Panels are structured identically to those in Figure 6, showing (**Left**) time series plots of actual (blue) and forecasted (red) CO values, and (**Right**) scatter plots comparing forecasted versus actual CO measurements. The diagonal lines, color coding, and accompanying histograms follow the same conventions as described in Figure 6, with σ_Forecasted_ representing the standard deviation of the forecasted CO values.

**Figure 9:**
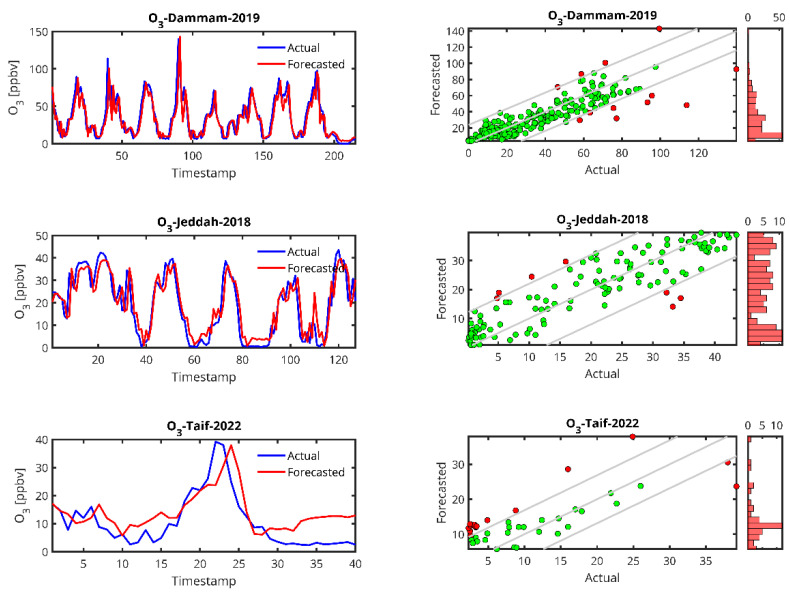
Actual and forecasted values for O_3_ concentrations. Panels are structured identically to those in Figure 6, showing (**Left**) time series plots of actual (blue) and forecasted (red) O_3_ values, and (**Right**) scatter plots comparing forecasted versus actual O_3_ measurements. The diagonal lines, color coding, and accompanying histograms follow the same conventions as described in Figure 6, with σ_Forecasted_ representing the standard deviation of the forecasted O_3_ values.

Figure 6, Figure 7, Figure 8 and Figure 9 show plots of the forecasted pollutants (PM_10_, PM_2.5_, CO and O_3_) as well as the scatter plots of the same pollutants for all the stations. It should be noted that in the scatter plot panels, the blue line represents the scatter plot of the ideal forecast for which the forecasted pollutant concentrations are equal to the actual concentrations. The red points denote the values of the concentrations forecasted by the LSTM algorithm vs. the actual concentrations. We draw the “Actual” values together with the “Forecasted” prediction to show dispersion of the actual measurements.

In the case of the PM_10_ prediction (Figure 6), we observe that LSTM can generally predict high peaks, i.e., the peak at timestamp 200 in the Jeddah 2019 data. We also observe that the lower the amount of data from the station, the higher the delay in the peak prediction and the lower the peak (Abba-2018, Riyadh-Aljazeera-2019–2020). The same trend is observed for PM_2.5_ (Figure 7), CO (Figure 8) and O_3_ (Figure 9) predictions.

By examining the results of the LSTM predictors (Table 4), PM_10_ in Jeddah (dataset collected for 2019), which has the largest dataset (2736 records), was found to outperform all other tested cities with a significantly higher R^2^ = 0.8029, lower MAPE = 25.7993 and an RMSE = 52.3117, indicating a much better model fit and prediction accuracy for PM_10_ in this city. However, the worst forecaster was for Riyadh (2019–2020) with a very high MAPE (49.9548), which indicates a poor model performance, and a negative (R^2^ = −0.3531) despite a moderate RMSE of 14.4530, although the RMSE values are within a reasonable range. This station is the one with the smallest dataset size. Also, Abha (2019) had relatively high MAPE values of 38.42 in 2018 and 35.85 in 2019, indicating that the LSTM model struggles more in predicting PM_10_ levels in this city. Overall, the performance of the LSTM predictor increases with the size of the dataset, as LSTM as a DL algorithm is known to be data demanding.

For PM_2.5_, although the results for the two cases studied are almost the same, a slight superiority was detected for the case of Rabigh station (R^2^ = 0.4926) when compared to Dhahban station (R^2^ = 0.4813). Both Jeddah (Rawda Stadium Station) and Jeddah (Dhahban Station) show high MAPE values (40.35 and 44.33), indicating significant prediction errors. The RMSE for Dhahban Station (2022) is relatively lower (8.99), suggesting that despite the larger percentage errors, the actual magnitude of the errors is smaller than for Rawda Stadium Station. Note that both sites are located in the province of Jeddah.

For CO, the performance metrics in terms of R^2^ range from 0.2445 (obtained in Abha) to 0.7735 (obtained in Dammam, 2019). Rabigh (2022) and Dammam (2019) show quite low MAPE values (4.34 and 5.58, respectively), indicating that the LSTM model accurately predicts CO levels in these regions. On the other hand, Jeddah (2019) has a remarkably high MAPE (50.48) and a relatively low R^2^ (0.43), indicating that the model struggles to predict CO concentrations despite a reasonable RMSE. Again, it can be seen that the performance is better for the case where the dataset size is relatively larger.

For O_3_, the two cases studied have comparable data records, which is reflected in the accuracy of the forecasts, which were found to be ~0.7 for R^2^ and 25 for MAPE. Jeddah (2018) and Dammam (2019) have low MAPE values (25.10 and 25.41) and high R^2^ values (0.81 and 0.77, respectively), indicating that the model performs well in predicting ozone concentrations in these cities. Dammam (2019) has a higher RMSE (12.89), but it is still within an acceptable range considering the complexity of the pollutant.

Some cities have particularly high MAPE values, such as Riyadh (2019–2020) for PM_10_ (49.95) and Jeddah (Rawda Stadium Station, 2022) for PM_2.5_ (40.35). These values indicate that the model predictions are less accurate in these locations and years. Possible reasons could be variations in pollution patterns, insufficient data, or the complexity of the underlying processes driving pollution levels. In contrast, the MAPE values for CO in Rabigh (2022) and Dammam (2019) are very low (4.34 and 5.57), indicating excellent model performance in these cities.

The R^2^ values in cities such as Jeddah (2019) for PM_10_ (0.80) and Jeddah (2018) for O_3_ (0.81) are high, indicating a good fit of the model to the data. This suggests that the model captures well the variance in pollutant concentrations in these areas. On the other hand, Riyadh (2019–2020) has no reported R^2^ value, and CO in Jeddah (2019) has a low R^2^ value (0.43), indicating poor model performance in these areas.

The RMSE values of CO in Rabigh (2022) show the lowest RMSE (0.0763), followed by Abha (2018) for PM_10_ (0.1204), indicating good overall prediction accuracy in terms of error size. On the contrary, PM_10_ in Jeddah (2019) (52.31) has the highest RMSE, indicating large prediction errors in terms of actual pollutant concentrations.

The size of the dataset plays a crucial role in the performance of the LSTM model. For example, larger datasets such as Jeddah (2019) (2736 records) for PM_10_ lead to better R^2^ (0.80) and lower MAPE (25.80), indicating that the model performs better when more data are available. In contrast, smaller datasets such as Riyadh (2019–2020) (92 records) have worse model performance MAPE (49.95), suggesting that the LSTM model may struggle to generalize well with fewer data points.

From the time series plots and the scatter plots shown in Figure 5, Figure 6, Figure 7 and Figure 8, the plots of the LSTM-predicted pollutant concentrations and the actual ones are almost similar, which is evidenced in the scatter plots where the distributions of the forecasted values are concentrated around the ideal scatter corresponding to the primary bisector line *y* = *x*. We observe that the higher the measurement, the fewer cases exist and at the same time, in general, the higher the real measurement, the higher the error, which could indicate that the scarcity of measurements in the high range hampers the LSTM training algorithm, pointing again to the high data requirement of the LSTM algorithm. According to the above temporal analysis of the results, the main finding confirms that LSTM is data hungry, which is reflected in the higher performance obtained with larger dataset sizes.

Regarding the regional distribution and since the main focus of our study was the prediction of spatial-temporal concentrations of pollutants, better forecasts were obtained in the case of densely populated cities (Jeddah as an example), cities with higher traffic as in the case of Mecca, where the transportation means as well as the movements of people are frequent for religious reasons (many Muslim pilgrims perform Umrah and Hajj in Mecca). In addition, the accuracy of the forecasts can be attributed to economic and industrial activities. For example, the city of Dammam (and surrounding cities such as Dhahran), located in the eastern region of Saudi Arabia, is known for its petrochemical activities, which affect the air quality by releasing pollutants (see the case of O_3_ in Dammam). Unfortunately, the case of the Saudi capital (Riyadh) has not been studied in detail due to the lack of sufficient data. In conclusion, the forecast accuracy varies by pollutant, city, and data availability; and the regional factors (population, traffic, industry) contribute to model success.

### 3.5. Weak Meteorological–Pollutant Links with Moderate Temperature Influence

As noted in several studies, the primary forcing parameters influencing air pollution levels are emissions and the meteorological properties of the atmosphere. For instance, meteorological parameters such as atmospheric pressure, wind speed and temperature have been found to impact PM_2.5_ concentration episodes in China [42]. Similarly, a study conducted in Sri Lanka [43] identified wind speed and direction as the most influential factors for pollutant concentrations, including PM_2.5_, PM_10_, CO and O_3_.

In our modeling approach, we also included, among other meteorological factors, both wind speed and wind direction as potential predictors to capture the dynamic dispersion and transport mechanisms of air pollutants. Wind speed affects the dilution and advection rates of pollutants, with higher speeds typically leading to faster dispersion and lower local concentrations. In contrast, wind direction is crucial for identifying upwind pollution sources and transboundary pollutant movement, particularly in urban areas where emissions may originate from neighboring industrial or transportation zones. The inclusion of these two variables was intended to account for spatial pollutant transport, even though our correlation analysis ultimately revealed their limited direct impact on predictive accuracy. Nevertheless, their presence ensures that the model remains physically interpretable and applicable in cases where meteorological conditions significantly influence air quality patterns.

**Figure 10:**
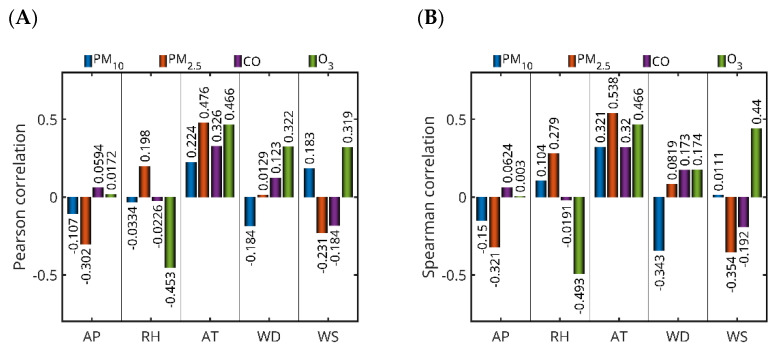
Bar plots of the Pearson (**A**) and Spearman (**B**) correlation coefficients between selected air pollutant concentrations of selected stations’ meteorological parameters—atmospheric pressure (AP), relative humidity (RH), ambient temperature (AT), wind direction (WD), and wind speed (WS). The pollutant datasets (D*) correspond to the best-performing LSTM forecasting models for the following: PM_10_ in Jeddah (2019), PM_2.5_ in Jeddah (Dhahban station, 2022), CO in Dammam (2019), and O_3_ in Jeddah (2018).

From this perspective, four additional experiments were carried out to investigate the effect of five meteorological parameters—ambient temperature (AT), atmospheric pressure (AP), wind speed (WS), wind direction (WD) and relative humidity (RH)—on the four studied pollutants (PM_2.5_, PM_10_, CO and O_3_) in Saudi Arabia. For this purpose, Pearson (linear) and Spearman (nonlinear) correlation coefficients were calculated between the pollutant concentrations from the best-performing models (which did not include meteorological parameters, as shown in Table 4) and the selected meteorological variables, in order to assess the strength of their relationships (Figure 10). Both Pearson and Spearman correlation coefficients yielded low values, indicating weak linear and nonlinear relationships, respectively, between the meteorological parameters and pollutant concentrations. Positive correlation values suggest that pollutant concentrations tend to increase with rising meteorological parameter values, whereas negative values imply a decrease.

For PM_10_ in Jeddah (2019), weak correlations were observed with all meteorological parameters. Notably, ambient temperature showed the strongest positive correlation among the variables (Pearson: 0.2242; Spearman: 0.3212), while wind direction exhibited a moderate negative Spearman correlation (−0.3434), suggesting some sensitivity of PM_10_ concentrations to both temperature and wind flow patterns. PM_2.5_ in Jeddah (Dhahban station, 2022) showed a stronger and more consistent relationship with meteorological conditions. Ambient temperature had a moderate positive correlation (Pearson: 0.4759; Spearman: 0.5383), while atmospheric pressure exhibited a moderate negative correlation (Pearson: −0.3023; Spearman: −0.3214). Wind speed was moderately negatively correlated (Spearman: −0.3545), indicating that higher wind speeds may contribute to pollutant dispersion. For CO in Dammam (2019), the most notable correlation was with ambient temperature (Pearson: 0.3259; Spearman: 0.3196), while all other parameters showed only weak associations. O_3_ in Jeddah (2018) displayed the strongest correlations with meteorological variables among all pollutants analyzed. A strong negative correlation was found with relative humidity (Pearson: −0.4527; Spearman: −0.4928), while ambient temperature (Pearson: 0.4658; Spearman: 0.4659) and wind speed (Pearson: 0.3187; Spearman: 0.4397) were positively correlated. These results are consistent with ozone’s photochemical formation process, which is known to intensify under higher temperatures and lower humidity.

Our results showed a negative correlation between WS and PM_2.5_, PM_10_, and CO levels, suggesting that lower wind speeds contribute to the accumulation of these pollutants. This relationship can be attributed to less horizontal spreading under calm atmospheric conditions, which minimizes dilution and transportation of air pollution from the source of emissions. Wind has an effective role in ventilating urban cities; when wind speeds are low, the spread of contaminants is suppressed, allowing accumulation near the ground surface. Moreover, temperature inversions that commonly occur in arid regions such as Saudi Arabia can trap air pollution in the boundary layer to suppress vertical diffusion. These findings are consistent with studies in other arid urban environments [44,45]. Overall, ambient temperature emerged as the most influential meteorological factor across all pollutants, with particularly strong associations for PM_2.5_ and O_3_. In contrast, atmospheric pressure and wind direction showed weaker and more variable correlations across pollutants and locations. These findings support the conclusion that meteorological parameters generally exert a weak to moderate influence on pollutant concentrations. Among the parameters analyzed, only ambient temperature demonstrated a consistently moderate correlation, while all other meteorological factors exhibited weak associations.

### 3.6. Ambient Temperature Negligibly Improves LSTM Accuracy

Since only ambient temperature showed a moderate correlation with pollutant concentration, we implemented four LSTM experiments aiming to forecast the pollutants’ concentrations as influenced by their historical lags and the historical and present values of the ambient temperature (AT). It should be noted that the four additional experiments focused solely on the best models for PM_10_, PM_2.5_, CO and O_3_. The main aim of this choice was to investigate whether the inclusion of the meteorological parameters could improve the forecasters’ accuracy. The results of the four additional experiments along with their corresponding models (without considering the ambient temperature) are provided in Table 5 below.

As shown in Table 5, the performance metrics of the LSTM-based pollutant forecasters using AT alongside historical pollutant data were quite similar to those from models relying solely on pollutant history.

**Figure 11:**
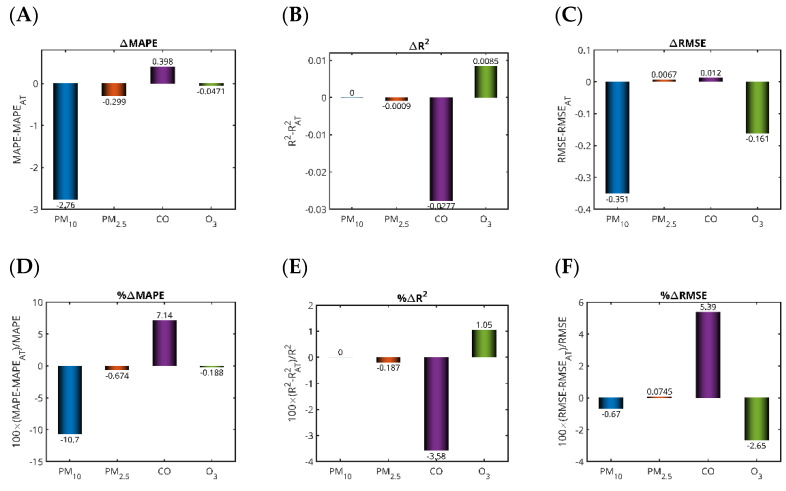
Differences in the performance metrics for the four additional experiments performed with the LSTM forecasters without the ones considering the ambient temperature as a meteorological predictor. The top row of panels shows the differences in MAPE (**A**), R^2^ (**B**) and RMSE (**C**), while the bottom row presents the corresponding percentages of the differences for MAPE (**D**), R^2^ (**E**) and RMSE (**F**). The pollutant datasets (D*) correspond to the best-performing LSTM forecasting models for the following: PM_10_ in Jeddah (2019), PM_2.5_ in Jeddah (Dhahban station, 2022), CO in Dammam (2019), and O_3_ in Jeddah (2018).

To evaluate the significance of these differences, we plotted bar charts comparing the differences (and performance) of the metrics obtained without and with AT as an input in the prediction model (Figure 11). Interestingly, including AT as an additional input variable in the LSTM model for CO pollution prediction proved beneficial to model performance, as indicated by the decrease in MAPE for CO (Figure 11A,D) and RMSE (Figure 11C,F) and the increase in R^2^ (Figure 11B,E). This outcome is likely due to the fact that temperature plays a significant role in the physical and chemical behavior of CO in the atmosphere. CO emissions are closely linked to combustion processes, which tend to produce more pollutants under lower temperature conditions due to incomplete combustion. Additionally, temperature influences atmospheric dynamics such as mixing layers and inversion conditions that can either disperse or trap pollutants near the surface. When the LSTM model is provided with temperature data alongside historical CO concentrations, it can better capture these complex, time-dependent interactions between environmental conditions and pollutant levels. This enriched input allows the model to learn more accurate patterns and relationships, leading to improved predictive capabilities.

In contrast, the inclusion of AT yielded a slight detrimental effect in the prediction of PM_10_ concentrations. This outcome is often attributed to the more complex and indirect relationship between temperature and PM_10_ concentrations compared to pollutants like CO. PM_10_ levels are influenced by a wide range of sources and processes, including vehicular emissions, industrial activities, dust resuspension, secondary particle formation, and meteorological conditions like wind speed, humidity, and precipitation. While temperature does play a role in atmospheric chemistry and dispersion, its effect on PM_10_ is more context-dependent and may not have a clear, consistent correlation across different scenarios and time periods. When temperature is included as an input, it can introduce additional noise or irrelevant patterns into the model. This could confuse the LSTM’s learning process, especially if the temperature–PM_10_ relationship is weak, nonlinear, or overshadowed by other dominant factors such as wind or humidity. The LSTM model, which is sensitive to the temporal structure and dependencies in the input data, may allocate part of its learning capacity to capture spurious or misleading patterns related to temperature, detracting from its ability to focus on more significant predictors. Consequently, the predictions can become less accurate, as indicated by increased error metrics like MAPE and RMSE, and a reduction in R^2^, reflecting poorer model fit and generalization. Moreover, in certain regions or seasons, higher temperatures might correlate with lower PM_10_ due to enhanced vertical mixing and dispersion, while in other contexts, such as during heatwaves or stagnant conditions, they might coincide with increased PM_10_ levels due to secondary aerosol formation. Such inconsistencies can make it challenging for the LSTM to learn a stable and generalizable relationship between temperature and PM_10_, leading to the model incorporating misleading associations that degrade overall performance.

From a logistical standpoint, the acquisition of additional measurements may impede the practical deployment of the model, as the integration of more sensors increases the system complexity and introduces a higher potential for failure due to sensor malfunction, calibration drift, or data inconsistency. From a computational perspective, introducing additional features (like the meteorological parameters) in the forecasters results in additional computing burden without clear improvement of the accuracy. Although it is important to consider the effect of other predictors, including the meteorological parameters, a trade-off between the gain in terms of accuracy and the complexity of the models should be expected. In the case of the Saudi Arabian context and based on the available datasets, it can be clearly concluded that adopting forecasters based only on the previous values of the pollutant concentration is sufficient to obtain reasonable accuracy while preventing additional computational burden.

In the context of Saudi Arabia, our findings revealed a relatively weak correlation between most meteorological parameters (e.g., humidity, pressure, wind speed, and wind direction) and pollutant concentrations. This outcome can be attributed to several interrelated factors. Saudi Arabia has an arid desert climate characterized by low humidity, infrequent rainfall, and relatively stable meteorological conditions, with minimal variation in climatic variables over time. These stable conditions limit the dynamic interaction between weather and pollutant dispersion or transformation, which may otherwise be observed in more variable climates (e.g., temperate or coastal regions). From a statistical perspective, when an external factor remains relatively constant, it tends to have little impact on variations in the dependent pollution variables. Another contributing factor may be the temporal and spatial resolution of the meteorological data used. These parameters were sourced from datasets that might not fully capture localized microclimatic variations, especially in complex urban environments. Additionally, the LSTM model architecture inherently captures temporal dependencies within the pollutant data, which may indirectly encode the effects of meteorological influences. Together, these reasons help explain why meteorological variables—except for ambient temperature, which showed a moderate effect—had limited influence on the predictive performance of the LSTM models.

### 3.7. Do LSTMs Outperform RF and XGBoost?

To further validate the performance of the proposed LSTM-based framework, we compared its predictive accuracy with two widely used ensemble-based ML algorithms: RF and XGBoost. Similarly to the effect of the temperature (Section 3.6), both models were tested on the same pollutant datasets (PM_10_, PM_2.5_, CO, and O_3_) for the four case studies that provided the best accuracy compared to their peers from the same category, using the same input structure and training/testing splits applied to the LSTM models. RF is a non-sequential ensemble learning method that builds multiple independent decision trees and averages their outputs to improve robustness and reduce overfitting. XGBoost, in contrast, uses a gradient boosting framework that builds trees sequentially, where each new tree corrects the errors made by the previous one. Both models are well-suited for structured tabular data and have shown competitive performance in various forecasting tasks. However, in contrast to LSTM, RF and XGBoost do not explicitly account for temporal dependencies between sequential data points. LSTM, a type of recurrent neural network, incorporates a memory cell structure and gating mechanisms that allow it to retain and update information across time steps. This ability to learn from historical patterns and long-term dependencies makes LSTM particularly effective for time-series forecasting tasks, such as pollutant concentration prediction. The results of eight experiments conducted under the same conditions as the LSTM are provided in Figure S1. Based on the results in Figure S1, the following comments regarding each of the four pollutants can be highlighted:

- PM_10_—Jeddah (2019): LSTM achieved a low MAPE (25.80%) and a strong R^2^ value (0.8029), outperforming RF in all metrics. However, XGBoost yielded the best overall performance with the highest R^2^ (0.8647) and the lowest MAPE (25.35%), although its RMSE remained higher than that of LSTM, suggesting occasional large errors. The RMSE for PM_10_ is higher than for the other pollutants because PM_10_ concentration values are, on average, much larger in magnitude. RMSE quantifies the typical size of prediction errors in the same units as the data, and since it scales directly with the magnitude of values, a pollutant like PM_10_—with its comparatively higher concentration levels—naturally yields a larger RMSE even if the relative forecast accuracy is similar.
- PM_2.5_—Jeddah (2022): All models performed relatively poorly due to the small dataset. LSTM produced a slightly better R^2^ (0.4813) and lower MAPE (44.33%) than RF and XGBoost. RF and XGBoost showed comparable results, but none of the models demonstrated robust predictive accuracy, indicating that data scarcity limited performance across all models.
- CO—Dammam (2019): XGBoost and RF outperformed LSTM, achieving lower MAPE and higher R^2^ values (0.8559 and 0.8497, respectively), despite higher RMSE values. This suggests that ensemble models may be more effective than LSTM in handling pollutant time series with less pronounced temporal dependencies or noise.
- O_3_—Jeddah (2018): LSTM provided the best trade-off with the lowest RMSE (6.08) and the highest R^2^ (0.8073), whereas RF and XGBoost both showed higher error levels and reduced predictive power, particularly XGBoost (R^2^ = 0.7725, RMSE = 15.43). This underscores the suitability of LSTM for ozone modeling in this context.

LSTM models consistently achieved superior or comparable performance across most pollutants, particularly when datasets were sufficiently large (e.g., PM_10_, O_3_). XGBoost performed best for PM_10_ and CO, indicating its strength in handling complex but short-term dependencies and structured features. RF generally underperformed compared to LSTM and XGBoost, except for CO, where it was competitive. Predictive accuracy across all models was notably impacted by dataset size, as seen with PM_2.5_, suggesting the need for larger or more diverse datasets for robust learning. For pollutants with clearer temporal patterns (O_3_, PM_10_), LSTM’s memory-based structure gave it a clear advantage, while RF and XGBoost were more effective in datasets where relationships were less temporally dependent (e.g., CO).

In conclusion, LSTM proved the most reliable model for time-series pollutant forecasting in this study, especially with large and clean datasets. However, XGBoost and RF offer competitive alternatives and may be preferable for certain pollutant types or data conditions. The observed superior performance of the LSTM model compared to RF and XGBoost can be attributed to its architectural advantages in handling sequential data. Unlike tree-based ensemble methods, LSTM is specifically designed to retain memory over long periods through input, forget and output gates, allowing it to detect hidden patterns and delayed effects—crucial in forecasting air pollution driven by multi-day accumulation or delayed meteorological and human influences. In contrast, RF and XGBoost rely on independent feature observations and may miss such dynamic dependencies. Our findings support the idea that LSTM’s ability to model sequential behaviors provides a clear advantage in temporal forecasting, especially in data-rich urban environments.

## 4. Discussion

This study highlights the importance of model selection based on urban density and data availability. It further outlines how model performance varies across cities and pollutants. A data-driven modeling approach based on three algorithms: LSTM, RF, and XGBoost, was carried out to predict the concentrations of various pollutants in Saudi Arabia. Datasets for key pollutants affecting human health (PM_10_, PM_2.5_, CO, and O_3_) were collected, analyzed, and visualized. The study has covered both the temporal (datasets from previous years) and spatial (many cities distributed over the Saudi Arabian territory) dimensions. Through trial and error, the crucial hyperparameters of the LSTM were optimally selected. The developed forecasters were then evaluated according to several performance metrics such as the coefficient of determination (R^2^). Accuracy ranged from moderate to good, with LSTM performing best when the dataset size was relatively large, confirming that DL algorithms are data-hungry. LSTM was also found to capture hidden features of the pollution data through the gating mechanisms. From a spatial perspective, LSTM gave better performance in densely populated cities and in cities with higher economic, transportation, and industrial activities. The performance of LSTM lies in its architecture, explicitly designed to handle sequential and time-dependent data.

In this study, we demonstrate that historical pollutant concentrations alone can be sufficient for accurate forecasting particularly when using the LSTM, which can capture nonlinear dependencies—unlike previous studies that relied heavily on meteorological variables. Through a detailed correlation analysis between pollutant concentrations and five meteorological variables (ambient temperature, pressure, humidity, wind speed, and wind direction), we found that, except for ambient temperature, which showed only a moderate effect, the remaining parameters had generally weak linear associations with pollutant levels. Interestingly, even when temperature was included, the predictive accuracy of models that considered it was similar to those that did not. While meteorological variables do influence the dispersion and concentration of air pollutants, air pollution processes themselves are governed by complex, nonlinear, and time-dependent interactions involving emissions, meteorological conditions, and chemical transformations. Our correlation analysis relied on linear measures that cannot fully capture these complexities; however, the LSTM model is specifically designed to learn nonlinear relationships and temporal dependencies. Despite the low linear correlations, the LSTM model achieved strong predictive performance across multiple evaluation metrics (e.g., RMSE, MAE, and R^2^), indicating that it successfully identified meaningful temporal patterns and complex dependencies beyond what linear correlation reveals. Moreover, meteorological factors may not exert an immediate effect but can influence pollutant levels with time lags, a dynamic the LSTM architecture can accommodate, unlike traditional correlation analysis. Additionally, pollutant concentrations are shaped not only by meteorological conditions but also by emission sources, land use, and chemical reactions, making low linear correlations with individual meteorological variables expected, especially when emissions are dominated by short-term variability. Overall, although direct linear correlations were low, the robust performance of the LSTM model underscores its strength in capturing the underlying nonlinear and lagged dynamics of air pollution. Consequently, it can be recommended that forecasting approaches relying solely on the historical values of pollutants themselves may be sufficient and effective for short-term prediction.

In this research, wind speed and wind direction were incorporated as predictors because of their role in dispersion and transport of air pollutants. Firstly, wind speed generally affects the dilution rate of pollutant concentration. The stronger the wind, the more the dispersion and the lower the local concentration. Conversely, the weaker the wind, the more there is accumulation of pollutants.

Additionally, wind direction shows an impact on the pollutant and further assists monitoring stations situated downwind from significant emission sources. Therefore, including both the wind speed and direction is important to improve the model and identify the relationship between pollution source and receptor, even when there is an absence of source data (i.e., traffic or emission interventions). Although these two variables did not show a significantly strong linear correlation with pollutant levels in our study, their non-linear effects may still enhance prediction accuracy in a model such as LSTM.

As a result, it can be recommended to adopt the forecasters based only on the historical (precedent) values of the pollutants themselves. In addition, RF and XGBoost have provided good results closely similar to those of the LSTM, with a superiority of the LSTM in capturing hidden relationships between factors.

It is well recognized that, in AI-based models, larger datasets generally enhance predictive performance by allowing the model to learn more comprehensive and representative patterns. However, in practical applications, particularly in environmental studies, the dataset size is often constrained by the availability and continuity of historical data collected under varying conditions. While there is currently no standardized method to equalize dataset sizes across different contexts, it remains essential to report and critically consider the dataset size when interpreting model outcomes. In this study, we utilized all the available data to ensure that our models were trained on the most complete information possible. This approach helps contextualize the observed predictive accuracy and underlines the importance of dataset completeness in environmental time series forecasting.

Additional data sources, such as traffic volume, land use pattern, and socioeconomic indicators, can further improve model performance, and additional future work can further explore the additional data sources. As a perspective, this study can be extended to larger datasets using other DL and swarm intelligence optimization techniques. Of particular interest could be the use of transformers that are more robust to the memory vanishing problem of the LSTM.

For regions with high MAPE values, it may be beneficial to focus on improving data preprocessing or enriching the dataset. The addition of external factors such as traffic data could potentially improve the models’ prediction accuracy. For cities with lower R^2^ values (such as Riyadh and Jeddah for CO), it may be valuable to consider adjusting the model architecture, tuning the hyperparameters, or using more advanced techniques such as feature engineering or ensemble models to improve predictions. Further studies could explore the sources of high prediction errors, focusing on some cities and pollutants and, in particular, investigating local environmental, meteorological, and socio-economic factors that may influence pollution levels and model predictions mainly for long-term forecasting contexts.

## 5. Conclusions

In this study, we explored the temporal and spatial dynamics of air pollution across Saudi Arabian cities and assessed the predictive performance of various AI models, including LSTM, RF, and XGBoost. Our findings highlight that LSTM models outperform ensemble ML methods, especially when an adequate data volume per monitoring station is available. The predictive capability of LSTM is closely linked to data quantity and urban density, while meteorological variables, except for ambient temperature, show limited influence on pollutant concentrations in this arid region. Importantly, adding temperature data into LSTM models yields only marginal gains, underscoring LSTM’s strength in capturing complex temporal dependencies from historical pollution data alone. These insights offer valuable guidance for policymakers and researchers aiming to enhance air quality forecasting in data-sparse environments, ultimately supporting public health and environmental decision-making in Saudi Arabia and similar regions.

## Figures and Tables

**Table 1:** Summary of selected studies on forecasting/modeling pollution indicators.

Ref.	Place	Period/ Dataset	Variables/Frequency	Methods	Results	Findings	Limitations
[26]	Salamanca, Mexico	NR	PM_10_/Hourly	Combination of MLP and clustering	Correlation from 0.51 to 0.77	Combining ANN with clustering improved accuracy	Limited accuracy
[27]	Seoul, Republic of Korea	NR	PM_10_, PM_2.5_	Tree-based ML	PM_10_ R^2^ = 0.86, PM_2.5_ R^2^ = 0.83	LGB outperforms other tree-based algorithms	Use assimilated data predicted to run forecasting
[28]	Various Stations, India	2017–2020	PM_10_, PM_2.5_, CO, NOX/Hourly	LSTM combined to GA	RMSE = 9.582	Tuning LSTM hyperparameters by GA improved the accuracy	High computation time since training LSTMs for multiple GA genes
[29]	Beijing, China	1 January 2010–/31 December 2014	PM_2.5_/Hourly	Bidirectional GRU DL	RMSE = 14.5319, MAE = 10.4798	Model error lower than other methods	Time-consuming training
[30]	23 Cities, India	1 January 2015–31 July 2020	PM_2.5_, PM_10_, NO, NO_2_, etc.	Several ML tested	MAE = 0.298, RMSE = 1.465	XGBoost the best among four algorithms	Difficulty handling missing data
[31]	Taiwan	2012–2018	PM_2.5_	Aggregated LSTM	RMSE = 3.94, MAE = 2.94	ALSTM outperformed LSTM and SVR	Model ignores external variables
[32]	Beijing, China	1 January 2014–31 December 2016 and 1 January 2017–31 December 2017	PM_2.5_, PM_10_, SO_2_, NO_2_, O_3_, CO	Wavelet decomposition with LSTM	MAPE = 7.45%	Model accurate and stable	Unclear hyperparameter selection
[33]	Southwestern US	1988–2020	Dust emissions (fine and coarse)/monthly	ML, MLR, SVM, RF, BRNN, Cubist	Fine: R = 0.67–0.80, RMSE = 0.40–0.52; Coarse: R = 0.69–0.73, RMSE = 2.01–2.34	Non-linear models best; fine dust more accurate; temp key predictor	Underestimated highs; coarse less accurate

Abbreviations: (ALSTM) Attention mechanism and Long Short-Term Memory, (ANN) Artificial Neural Network, (BRNN) Bayesian Regularized Neural Networks, (DL) Deep Learning, (GA) Genetic Algorithm, (GRU) Gated Recurrent Unit, (LGB) Light Gradient Boosting, (MAPE) Mean Absolute Percentage Error, (ML) Machine Learning, (MLR) Multiple Linear Regression, (NR) Not Reported, (Ref.) Reference, (RF) Random Forests, (SVM) Support Vector Machines, (SVR) Support Vector Regression, (XGBoost) eXtreme Gradient Boosting.

**Table 2:** Description of the main features of the datasets used. PM_10_ and PM_2.5_ are measured in µg m^−3^, the CO and O_3_ in parts per billion by volume (ppbv).

Pollutant	City	Frequency	Start Date/Time	End Date/Time	Dataset Size	Comment
PM_10_	Abha	Hourly	04/04/2018-21:00	18/04/2018-20:00	336	Consecutive regularly spaced
	Abha	Hourly	01/01/2019-00:00	28/01/2019-10:00	658	Consecutive regularly spaced
	Jeddah	Hourly	01/01/2019-01:00	25/04/2019-00:00	2736	Consecutive regularly spaced
	Mecca	Hourly	01/01/2021 1:00 AM	11/02/2021-4:00 PM	1000	Consecutive regularly spaced from Al-Haram station
	Rabigh	Hourly	01/10/2021-1:00:00 AM	02/07/2021-1:00:00 PM	901	Consecutive regularly spaced
	Riyadh (Al-Jazeera Station)	Hourly average/daily	01/08/2019	09/07/2020	92	Consecutive not regularly spaced
PM_2.5_	Jeddah (Rawda Stadium Station)	Hourly	02/01/2022 9:00	01/05/2022 14:00	644	Consecutive regularly spaced
	Jeddah (Dhahban Station)	Hourly	21/01/2022 8:00	01/02/2022 10:00	455	Consecutive regularly spaced
CO	Rabigh	Hourly	01/01/2022 1:00 AM	02/11/2022 4:00 PM	1000	Consecutive regularly spaced
	Jeddah	Hourly	01/01/2019 1:00 AM	25/01/2019- 4:00 PM	580	Consecutive regularly spaced with missing values
	Dammam	Hourly	04/04/2019 9:00	28/02/2019 16:00	834	Consecutive regularly spaced
	Abha	Hourly	01/01/2018 00:00	09/01/2018 9:00	202	Consecutive regularly spaced
O_3_	Jeddah	Hourly	02/07/2018 23:00	01/12/2018 11:00	637	Consecutive regularly spaced
	Dammam	Hourly	06/02/2019 20:00	19/04/2019 01:00	1076	Consecutive regularly spaced
	Taif	Hourly	01/01/2022 1:00:00 AM	01/09/2022 10:00:00 AM	202	Consecutive regularly spaced

**Table 3:** LSTM hyperparameter tuning using the trial-and-error method.

Hyperparameter #	Name	Value	Comment
1	Scaler	MinMax	Between −1 and 1
2	Sequence length	5	Reasonable choice using trial-and-error
3	Training dataset	0.8	80% of the dataset was used for training (in-sample)
4	Testing dataset	0.2	20% of the dataset was used for the model testing (out-of-sample)
5	LSTM number of units	200	Adopted after many trials
6	Activation function	Rectified Linear Unit (ReLu)	It exhibits faster computation over other activation functions
7	Dense layer units	1	Commonly used
8	Training loss function	Mean-squared-error (MSE)	-
9	Optimizer	Adam	-
10	Batch size	64	-

**Table 4:** Performance metrics of the experiments performed with the LSTM predictors. The best cases are highlighted in bold, and the worst cases are highlighted in italics.

Pollutant	City	Dataset Size	Testing Dataset Size	MAPE	R^2^	RMSE
PM_10_	Abha (2018)	336	67	38.42	0.3302	17.25
	Abha (2019)	658	131	35.85	0.3031	16.39
	Jeddah (2019)	2736	547	25.80	0.8029	*52.31*
	Mecca (2021)	1000	199	28.22	0.6474	35.75
	Rabigh (2021)	901	181	21.55	0.5947	19.62
	Riyadh (Al-Jazeera station) (2019–2020)	92	18	49.95	*−0.3531*	14.45
PM_2.5_	Jeddah (Rawda Stadium station) (2022)	644	128	40.35	0.4926	32.38
	Jeddah (Dhahban station) (2022)	455	90	44.33	0.4813	8.988
CO	Rabigh (2022)	1000	199	**4.342**	0.7195	**0.0763**
	Jeddah (2019)	580	115	*50.48*	0.4261	0.2992
	Dammam (2019)	834	166	5.578	0.7735	0.2227
	Abha (2018)	202	40	12.50	0.2445	0.1204
O_3_	Jeddah (2018)	637	127	25.09	**0.8073**	6.079
	Dammam (2019)	1076	215	25.41	0.7669	12.89
	Taif (2019)	202	40	53.92	0.4514	6.891

**Table 5:** Performance metrics of the four additional experiments performed with the LSTM forecasters without and with consideration of the ambient temperature (AT) as a meteorological predictor. The best cases are highlighted in bold, and the worst cases are highlighted in italics.

Pollutant	Use AT	City	Dataset Size	Testing Dataset Size	MAPE	R^2^	RMSE
PM_10_	No	Jeddah (2019)	2736	547	25.80	0.8029	*52.31*
	Yes				28.56	0.8001	52.66
PM_2.5_	No	Jeddah (Dhahban station) (2022)	455	90	44.33	*0.4813*	8.988
	Yes				*44.63*	0.4822	8.981
CO	No	Dammam (2019)	834	166	5.578	0.7735	0.2227
	Yes				**5.18**	0.8012	**0.2107**
O_3_	No	Jeddah (2018)	637	127	25.10	**0.8073**	6.080
	Yes				25.14	0.7988	6.2416

## Data Availability

Data will be made available on reasonable request.

## Supplementary Materials

The following supporting information can be downloaded at: https://www.mdpi.com/article/10.3390/toxics13080682/s1, Figure S1: Performance metrics for the comparison experiments between the LSTM, RF and XGBoost forecasters. (A) MAPE, (B), R^2^ and (C) RMSE. The pollutant datasets (D*) correspond with the best-performing LSTM forecasting models for the following: PM_10_ in Jeddah (2019), PM_2.5_ in Jeddah (Dhahban station, 2022), CO in Dammam (2019), and O_3_ in Jeddah (2018).
