# Supplementary material for: Spatial-Temporal Forecasting of Air Pollution in Saudi Arabian Cities Based on a Deep Learning Framework Enabled by AI
Source: Toxics. 2025 Aug 16;13(8):682. doi: 10.3390/toxics13080682 (PMC12390308; doi:10.3390/toxics13080682)
Supplement: Supplementary file 1 [file toxics-13-00682-s001.zip › toxics-3767146-supplementary.pdf]

toxics

MDPI

Supplementary Material

Article

Spatial-Temporal Forecasting of Air Pollution in Saudi Arabian Cities Based on a Deep Learning Framework Enabled by AI

Rafat S. Zrieq [1,2], Souad Kamel [3], Faris Al-Hamazani [4], Sahbi Boubaker [3], Rozan Attili [5] and Marcos J. Araúzo-Bravo [6,7,8,*]

1 Department of Public Health, College of Public Health and Health Informatics, University of Ha'il, Ha'il 55471, Saudi Arabia; r.zrieq@uoh.edu.sa (R.Z.)
2 Applied Science Research Center, Applied Science Private University, Amman 11937, Jordan
3 Department of Computer & Network Engineering, College of Computer Science and Engineering, University of Jeddah, Jeddah 21959, Saudi Arabia; skamel@uj.edu.sa (S.K.); sboubaker@uj.edu.sa (S.B.)
4 Department of Health Informatics, College of Public Health and Health Informatics, University of Ha'il, Ha'il 55471, Saudi Arabia; f.alhamzani@uoh.edu.sa
5 Department of Medical Laboratory Science, Faculty of Pharmacy and Medical Science, Hebron University, Hebron P700, Palestine; rozana@hebron.edu
6 Computational Biology and Systems Biomedicine, Biogipuzkoa Health Research Institute, San Sebastian 20014, Spain
7 Basque Foundation for Science, IKERBASQUE, Bilbao 48009, Spain
8 Department of Cell Biology and Histology, Faculty of Medicine and Nursing, University of Basque Country (UPV/EHU), Leioa 48940, Spain
* Correspondence: mararabra@yahoo.co.uk

Supplementary figures

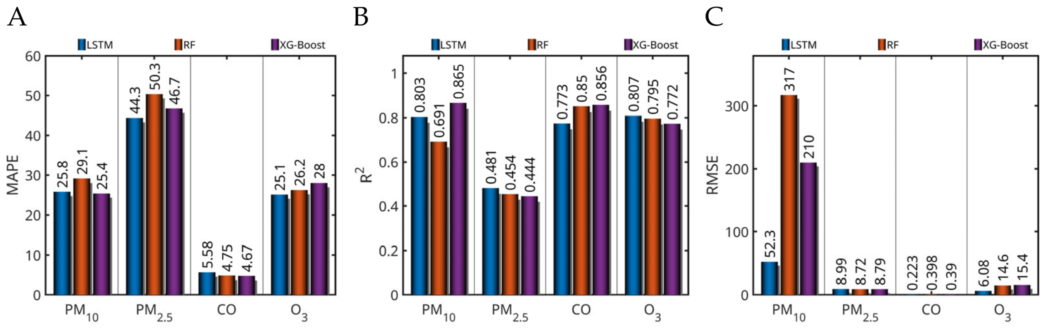

Figure S1. Performance metrics for the comparison experiments between the LSTM, RF and XGBoost forecasters. (A) MAPE, (B), R^2 and (C) RMSE. The pollutant datasets (D*) correspond with the best-performing LSTM forecasting models for the following: PM_{10} in Jeddah (2019), $PM_{2.5}$ in Jeddah (Dhahban station, 2022), CO in Dammam (2019), and O_3 in Jeddah (2018).
